# Impact of quality improvement strategies on the quality of life and well-being of individuals with spinal cord injury: a systematic review protocol

**DOI:** 10.1186/2046-4053-2-14

**Published:** 2013-02-22

**Authors:** Sarah EP Munce, Laure Perrier, Andrea C Tricco, Sharon E Straus, Michael G Fehlings, Monika Kastner, Eunice Jang, Fiona Webster, Susan B Jaglal

**Affiliations:** 1 Institute of Health Policy, Management & Evaluation, University of Toronto, 160-500 University Ave, M5G 1V7, Toronto, ON, Canada; 2 Li Ka Shing Knowledge Institute of St. Michael’s Hospital, Toronto, ON, Canada; 3 Division of Neurosurgery, Department of Surgery, University of Toronto, Toronto, ON, Canada; 4 Department of Applied Psychology & Human Development Ontario Institute for Studies in Education, Toronto, ON, Canada; 5 Department of Family and Community Medicine, University of Toronto, Toronto, ON, Canada; 6 Department of Physical Therapy, University of Toronto, Toronto, ON, Canada

**Keywords:** Quality of life, Well-being, Spinal cord injury, Quality improvement, Systematic review, Protocol

## Abstract

**Background:**

After a spinal cord injury, quality of life, as well as the determinants of quality of life, has been widely assessed. However, to date, there have been no systematic reviews on the impact of quality improvement strategies, including self-management strategies, on the quality of life and well-being of individuals with a spinal cord injury. The current protocol outlines a strategy for a systematic review that aims to identify, assess, and synthesize evidence on the impact of quality improvement strategies on the quality of life and physical and psychological well-being of individuals with spinal cord injury.

**Methods/Design:**

All study designs, except qualitative studies will be included. Studies reporting on quality improvement including audit and feedback, case management, team changes, electronic patient registries, clinician education, clinical reminders, facilitated relay of clinical information to clinicians, patient education, (promotion of) self-management, patient reminder systems, and continuous quality improvement among individuals with spinal cord injury will be included. The primary outcome is quality of life. The secondary outcomes are physical and psychological well-being. Studies will be included regardless of publication status, year of dissemination, or language of dissemination. Potentially relevant articles not written in English will be translated. We will search Medline, CINAHL, EMBASE, and PsycINFO. The use of these databases will be supplemented by other data sources, including unpublished data. Two independent reviewers will conduct all levels of screening, data abstraction, and quality appraisal. Results will be grouped according to the target group of the varying quality improvement strategies (that is, health system, health-care professionals, or patients) and/or by any other noteworthy grouping variable, such as etiology of spinal cord condition or by sex. If deemed appropriate, a meta-analysis will be conducted.

**Discussion:**

This systematic review will identify those quality improvement strategies aimed at the health system, health-care professionals, and patients that impact the quality of life and well-being of individuals with spinal cord injury. Knowledge and application of such quality improvement strategies may reduce inappropriate health-care utilization costs, such as acute care inpatient readmission in the years post injury. Prospero registry number: CRD42012003058.

## Background

A spinal cord injury (SCI) results in a number of motor, sensory, and autonomic impairments. It predisposes individuals to multisystem dysfunction, leading to an increased likelihood of a range of related secondary complications [1-4], defined as medical consequences that can cause functional limitations. Common secondary health complications after SCI include pressure ulcers, urinary tract infections, bowel problems, fractures, chronic pain, and depressive disorders [5]. Despite the fact that many of these complications are amenable to treatment and/or prevention, secondary complications represent a significant burden at both the health system and individual level [6-8].

As a result of secondary complications, individuals with a SCI have greater rates of contact with the health-care system than the general population, and also have multiple rehospitalizations throughout their lifetime. For example, Dryden and colleagues (2004) [9] found that compared with a control group, individuals with a SCI required 30 more hours of home-care services, were 2.7 times more likely to have physician contact, spent 3.3 more days in hospital, and were rehospitalized 2.6 times more often. Rehospitalization following SCI has been studied in a number of countries including the United States (US), Britain, Australia [10], the Netherlands [11], Italy [12], and Turkey [4]. These studies have reported that approximately one-third of persons with a traumatic SCI will be rehospitalized each year [13]. More recently, our team reported a similar readmission rate of 27.5% one year after initial acute care discharge among individuals with traumatic SCI in Ontario. Secondary complications, including musculoskeletal, respiratory, gastrointestinal, and urological disorders, were the main reasons for readmission [14]. A large number of visits to family physicians and physiatrists has also been reported [15]. We concluded that the high rate of physician and specialist utilization, emergency department visits, and hospital readmissions, indicate that current care practices are not managing or preventing secondary complications adequately. We suggested that future research is required on strategies that can be implemented to improve the long-term quality of care for individuals with traumatic SCI [14,15].

At the individual level, secondary complications related to a SCI also intensify the experience of disability for people with a SCI by negatively impacting on quality of life including long-term health, productivity/employment, social participation, dignity, mobility, and independence [7]. People with a SCI tend to report fewer feelings of well-being, on average, than non-disabled persons; score lower on physical, mental, and social health, and in other domains of life that people consider important to life quality [16-18]. Thus, quality of life and well-being, and their determinants, have become important outcomes in SCI research and have been widely assessed [19-21]. In a recent systematic review of associations between psychological factors and quality of life, van Leeuwen and colleagues (2012) [22] determined that self-efficacy and self-esteem are consistently related to a better quality of life. As such, they suggested that self-management strategies, counseling, or cognitive behavioral therapy may be useful approaches for improving quality of life in this population. However, to date, no systematic reviews exist on the impact of quality improvement (QI) strategies, (including self-management strategies), on the quality of life and the physical and psychological well-being of individuals with an SCI. Thus, the current protocol outlines a strategy for a systematic review that aims to identify, assess, and synthesize evidence on the impact of QI strategies on the quality of life and the physical and psychological well-being of individuals with a SCI.

## Methods/Design

This protocol is informed by the guidelines from The Cochrane Collaboration [23] and the final report will conform to the Preferred Reporting Items for Systematic Reviews and Meta-Analyses [24]. This protocol was registered with the Prospero database (registration number CRD42012003058).

### Eligibility criteria

Studies will be eligible if they examine a predefined list of QI strategies for adults (≥18 years of age) with a spinal cord injury. Quality improvement strategies will include those targeted at health systems (for example, team changes), health-care professionals (for example, professional reminders), or patients (for example, reminders). Specific strategies will include audit and feedback, case management, team changes, electronic patient registries, clinician education, clinical reminders, facilitated relay of clinical information to clinicians, patient education, (promotion of) self-management, patient reminder systems, and continuous QI. This list of QI strategies has been previously identified through other systematic reviews and meta-analyses as important (namely, Shojania *et al*., 2006; Tricco *et al*., 2012) [25,26]. Outcomes of interest will be quality of life (primary), and physical and psychological well-being (secondary). For the purposes of this review, quality of life is operationalized as an individual’s perception of his or her position in life in the context of the culture and value systems in which he or she lives and in relation to his or her goals, expectations, standards, and concerns [27]. Well-being is defined as 1) a subjective or objective perception of improvement in physical health or of symptoms related to SCI or to side effects of treatment; and/or 2) a subjective or objective perception of improvement of psychological functioning [27]. To be included in the analysis, studies must report on quality of life as measured by validated scales, classifications, and measurement systems (for example, the Short-Form-36 (SF-36) or the Individual Quality of Life Interview). To capture physical well-being, studies reporting on the occurrence or severity of secondary complications, including autonomic dysreflexia, pressure ulcers, urinary tract infections, pneumonia, hypotension, bowel problems, deep vein thrombosis (in the legs or lungs), fractures, and chronic pain [5], will be included. Since health-care utilization, including physician and specialist utilization, emergency department visits, and hospital readmissions, is often associated with these secondary complications [14,15], we will also include studies that report on these outcomes. Finally, studies will be included if they report on psychological well-being as measured by validated and specific standardized impairment, distress, or psychological scales (for example, the Center for Epidemiologic Studies Depression Scale (CESD) [28] or the Hospital Anxiety and Depression Scale (HADS) [29]). Our systematic review will include experimental studies (including randomized controlled trials, quasi-randomized trials, and controlled clinical trials), quasi-experimental studies (including interrupted time series and controlled before and after studies), and observational studies. Studies will be included regardless of publication status, year of dissemination, or language of dissemination. Potentially relevant articles not written in English will be translated. However, a bias towards published studies and English-language materials may still be likely.

### Information sources and literature search

Literature search strategies will be developed using medical subject headings (MeSH) and text words related to QI strategies in the management of spinal cord injury. Studies will be identified by searching Medline (OVID interface, 1947 onwards), CINAHL (EBSCO interface, 1981 onwards), EMBASE (OVID interface, 1946 to present), and PsycINFO (OVID interface, 1806 onwards). The search strategy for Medline can be found in Additional file 1. In addition to the electronic databases, grey literature (that is, unpublished and difficult to locate material) will be searched. Unpublished material will be identified by searching the websites of relevant organizations (for example, Christopher & Dana Reeve Foundation, Rick Hansen Institute), the Dissertations and Theses database, and searching for relevant abstracts from conference proceedings via the Conference Papers Index (for example, the International Spinal Cord Society’s conferences). A hand search of the reference lists from 1990 to the present from reviews and selected articles from relevant journals (for example, *Spinal Cord*, *Disability & Rehabilitation*, and *Journal of Healthcare Management*) will be made to ensure a complete search. Finally, experts in the field of SCI (including one of the authors, MGF) will be contacted and consulted in order to ensure that all relevant data is obtained. An experienced information specialist (LP) will conduct all of the literature searches.

### Study selection process

To increase the reliability of screening by the reviewers, a pilot test of a predefined screening form based on the eligibility criteria described above (that is, section 2.1) on a random 1% sample will be performed prior to this process in order to increase reliability. The kappa statistic will be calculated to determine the inter-rater agreement for study inclusion [30]. If necessary, the inclusion and exclusion criteria will be clarified to promote the consistent application of the selection criteria (for example, to ensure the reviewers are aware of what qualifies as a QI strategy). Two reviewers will independently screen the titles and abstracts identified by the literature search for inclusion using the screening form (level 1 screening). The full text of potentially relevant articles will then be obtained and screened to determine final inclusion (level 2 screening). Discussion or the involvement of a third reviewer who is knowledgeable in the research area will be available to resolve discrepancies. Studies excluded during the screening phase will be recorded along with the reason(s) for exclusion.

### Data items and data collection process

Abstracted data will include study characteristics (for example, author names, year of publication, country of study, study design, and sample size), participant characteristics (for example, etiology of SCI (that is, traumatic or non-traumatic), mean age, and level of impairment), and outcome results (for example, specific scale/measure of quality of life, specific scale/measure of psychological well-being, measurement of depressive symptoms, and social support).

As in the study selection process, a data abstraction form will be pilot tested, standardized, and modified if poor agreement is observed. For example, any wording on the form that may be contributing to poor agreement will be reviewed and modified, as necessary. Two reviewers will independently abstract all of the data, and a discussion or the involvement or a third reviewer will resolve discrepancies.

### Methodological quality/risk of bias appraisal

Standardized quality assessment tools will be used to determine the methodological quality and the risk of bias of the included studies. We will use the Cochrane Risk of Bias Tool for randomized controlled trials [23]; the Cochrane Effective Practice and Organisation of Practice Risk of Bias Tool [31] for controlled clinical trials, interrupted time series, and controlled before-after studies; and the Newcastle-Ottawa Scale [32] for cohort studies and case control studies.

### Synthesis of included studies

The results of the systematic review will be summarized descriptively. Results will be grouped according to the target group of the varying QI strategies (that is, health system, health-care professionals, or patients) and/or by any other noteworthy grouping variable (for example, by etiology of spinal cord condition and/or by sex). If low statistical (for example, *I*^2^ < 60%) [33], methodological, and clinical heterogeneity is observed, a random effects meta-analysis will be conducted [34]. The mean difference will be used for continuous outcomes (for example, the SF-36 quality of life scale) and the relative risk will be used for dichotomous outcomes (for example, the Composite International Diagnostic Interview Short Form (CIDI-SF) [35]; depression (yes/no)). All analyses will be conducted in Review Manager Version 5.1 (available at http://ims.cochrane.org/revman).

## Discussion

An appropriate knowledge translation strategy will be implemented at the conclusion of the review. For example, the results of the systematic review will be presented at relevant meetings locally, nationally (for example, at the National Spinal Cord Injury Conference), and internationally (for example, at the International Spinal Cord Society Conference) and published in a peer-reviewed journal so that results are available to the appropriate academic and clinical audiences. The findings will also be disseminated through the newsletters (print and on-line) of interested organizations, such as the Ontario Neurotrauma Foundation and Spinal Cord Injury Canada. Lastly, partnerships with local clinical programs (for example, rehabilitation programs) and/or research initiatives (for example, the Participation and Quality of Life Toolkit) will be made to give timely and effective application of the research results.

This systematic review will identify those QI strategies aimed at the health system, health-care professionals, and patients that impact the quality of life and the physical and psychological well-being of individuals with a spinal cord injury. Knowledge and application of such QI strategies may reduce inappropriate health-care utilization costs, such as acute care inpatient readmission [36], in the years post injury.

## Abbreviations

MeSH: Medical subject heading; QI: Quality improvement; SCI: Spinal cord injury

## Competing interests

The authors declare they have no competing interests.

## Authors’ contributions

SEPM, LP, ACT, SES, and SBJ contributed to the conception and design of the review. LP developed the search strategies. SEPM, LP, ACT, SES, and SBJ provided feedback on the protocol during its development. SEPM wrote the first draft, which was revised by LP, ACT, SES, MGF, MK, EJ, FW, and SBJ. SEPM registered the protocol. All authors read and approved the final draft.

## Supplementary Material

Additional file 1 Ovid Medline (R) <1946 to July Week 2, 2012>, Ovid Medline (R) In-Process & Other Non-Indexed Citations <July 23, 2012>. (2011–2013). Click here for file
