# Supplementary material for: Impact of quality improvement strategies on the quality of life and well-being of individuals with spinal cord injury: a systematic review protocol
Source: Syst Rev. 2013 Feb 22;2:14. doi: 10.1186/2046-4053-2-14 (PMC3599324; doi:10.1186/2046-4053-2-14)
Supplement: Additional file 1 — Ovid Medline (R) <1946 to July Week 2, 2012>, Ovid Medline (R) In-Process & Other Non-Indexed Citations . (2011–2013). [file 2046-4053-2-14-S1.pdf]

Database: Ovid MEDLINE(R) <1946 to July Week 2 2012>, Ovid MEDLINE(R) In-Process & Other Non-Indexed Citations <July 23, 2012>

Search Strategy:

----

1 "Continuity of Patient Care"/ [QI strategies]

2 Case Management/

3 Disease Management/

4 "Delivery of Health Care, Integrated"/

5 Progressive Patient Care/

6 "Patient-Centered Care"/

7 "Patient Care Planning"/

8 (case adj coordination).tw.

9 (case adj co-ordination).tw.

10 (case adj management).tw.

11 (care adj management).tw.

12 (coordinat$ adj1 care).tw.

13 (co-ordinat$ adj1 care).tw.

14 (continu$ adj1 care).tw.

15 "continuity of care".tw.

16 comanag$.tw.

17 co-manag$.tw.

18 (care adj3 aftercare).tw.

19 (care adj3 after-care).tw.

20 (care adj3 collaborat$).tw.

21 (care adj3 cooperat$).tw.

22 (care adj3 co-operat$).tw.

23 (care adj3 interinstitution$).tw.

24 (care adj3 inter-institution$).tw.

25 (care adj3 progressive).tw.

26 (care adj3 shared).tw.

27 (care adj3 transition$).tw.

28 (disease adj management).tw.

29 (disconnect$ adj2 care).tw.

30 "discontinuity of care".tw.

31 discoordinat$.tw.

32 disco-ordinat$.tw.

33 (fragment$ adj care).tw.

34 (gap? adj2 care).tw.
35 handover$.tw.
36 (hand adj over$).tw.
37 (harmon$ adj care).tw.
38 (integrat$ adj2 care).tw.
39 (lia?son adj service?).tw.
40 "patient care plan$".tw.
41 ("patient centred" adj2 care).tw.
42 ("patient centered" adj2 care).tw.
43 (shared adj care).tw.
44 (seamless adj care).tw.
45 (synchron$ adj2 care).tw.
46 (transition$ adj care).tw.
47 (uncoordinat$ adj care).tw.
48 (unco-ordinat$ adj care).tw.
49 Medical Records Systems, Computerized/ [clinical information systems]
50 "clinical information system?".tw.
51 "clinical data system?".tw.
52 "information retrieval system?".tw.
53 "ancillary information system?".tw.
54 Decision Support Techniques/ [decision support]
55 Decision Support Systems, Clinical/
56 Decision-Making Computer Assisted/
57 "computer$ decision making".tw.
58 (decision$ adj2 support$).tw.
59 (decision$ adj2 aid$).tw.
60 (decision$ adj2 tool$).tw.
61 (decision$ adj2 instrument$).tw.
62 (decision$ adj2 technolog$).tw.
63 (decision$ adj2 technique$).tw.
64 (decision$ adj2 system$).tw.
65 (decision$ adj2 program$).tw.
66 (decision$ adj2 algorithm$).tw.
67 (decision$ adj2 process$).tw.
68 (decision$ adj material$).tw.
69 (decision$ adj2 counsel$).tw.
70 (decision$ adj2 guid$).tw.

71 (decision$ adj board).tw.

72 (interacti$ adj3 tool$).tw.

73 "risk communication tool$".tw.

74 interprofession$.tw. [team changes]

75 inter-profession$.tw.

76 interdisciplin$.tw.

77 inter-disciplin$.tw.

78 interoccupation$.tw.

79 inter-occupation$.tw.

80 interdepartment$.tw.

81 inter-department$.tw.

82 Interprofessional Relations/

83 team$.tw.

84 multiprofession$.tw.

85 multi-profession$.tw.

86 multidisciplin$.tw.

87 multi-disciplin$.tw.

88 multioccupation$.tw.

89 multi-occupation$.tw.

90 Patient Participation/ [self management]

91 Patient Education as Topic/

92 Self Care/

93 (client? adj1 train$).tw.

94 (client? adj1 educat$).tw.

95 (client? adj1 teach$).tw.

96 (client? adj1 instruct$).tw.

97 (consumer? adj1 train$).tw.

98 (consumer? adj1 educat$).tw.

99 (consumer? adj1 teach$).tw.

100 (consumer? adj1 instruct$).tw.

101 (patient? adj1 train$).tw.

102 (patient? adj1 educat$).tw.

103 (patient? adj1 teach$).tw.

104 (patient? adj1 instruct$).tw.

105 self-manag$.tw.

106 (self adj manag$).tw.

107 self-car$.tw.

108 (self adj car$).tw.
109 self-monitor$.tw.
110 (self adj monitor$).tw.
111 self-administrat$.tw.
112 (self adj administrat$).tw.
113 self-medicat$.tw.
114 (self adj medicat$).tw.
115 Remote Consultation/ [telemonitoring]
116 exp Telemedicine/ and home$.tw.
117 exp Telemedicine/ and in-home$.tw.
118 Telehealth/ and home$.tw.
119 Telehealth/ and in-home$.tw.
120 telemonitor$.tw.
121 tele-monitor$.tw.
122 telehome$.tw.
123 tele-home$.tw.
124 (home$ and tele$).tw.
125 (in-home$ and tele$).tw.
126 (home$ and (remote adj2 monitor$)).tw.
127 (in-home$ and (remote adj2 monitor$)).tw.
128 (home$ and (wireless adj2 monitor$)).tw.
129 (in-home$ and (wireless adj2 monitor$)).tw.
130 (home$ and (mobile adj2 monitor$)).tw.
131 (in-home$ and (mobile adj2 monitor$)).tw.
132 (home$ and (cell$ adj2 monitor$)).mp.
133 (in-home$ and (cell$ adj2 monitor$)).mp.
134 (home$ and (remote adj2 consult$)).tw.
135 (in-home$ and (remote adj2 consult$)).tw.
136 (home$ and (wireless adj2 consult$)).tw.
137 (in-home$ and (wireless adj2 consult$)).tw.
138 (home$ and (mobile adj2 consult$)).tw.
139 (in-home$ and (mobile adj2 consult$)).tw.
140 (home$ and (cell$ adj2 consult$)).mp.
141 (in-home$ and (cell$ adj2 consult$)).mp.
142 or/1-141
143 exp Spinal Cord Injuries/ [SCI]
144 exp Spinal Cord Ischemia/

145 exp Spinal Cord/
146 exp Central Cord Syndrome/
147 exp Cervical Vertebrae/in [Injuries]
148 exp Paraplegia/
149 exp Quadriplegia/
150 "central cord injury syndrome?".tw.
151 "central spinal cord syndrome?".tw.
152 (myelopathy adj3 traumatic).tw.
153 (myelopathy adj3 post-traumatic).tw.
154 paraplegi$.tw.
155 quadriplegi$.tw.
156 tetraplegi$.tw.
157 (spine adj2 fracture$).tw.
158 (spine adj2 wound$).tw.
159 (spine adj2 trauma$).tw.
160 (spine adj2 injur$).tw.
161 (spine adj2 damage?).tw.
162 (spine adj2 lesion$).tw.
163 (spinal adj2 fracture$).tw.
164 (spinal adj2 wound$).tw.
165 (spinal adj2 trauma$).tw.
166 (spinal adj2 injur$).tw.
167 (spinal adj2 damage?).tw.
168 (spinal adj2 lesion$).tw.
169 ("spinal cord" adj contusion$).tw.
170 ("spinal cord" adj laceration$).tw.
171 ("spinal cord" adj transaction$).tw.
172 ("spinal cord" adj trauma$).tw.
173 ("spinal cord" adj ischemia$).tw.
174 SCI.tw.
175 or/143-174
176 142 and 175
177 exp Animals/ not (exp Animals/ and Humans/)
178 176 not 177
